# Health beliefs and quality of life in patients with gastric cancer: the chain mediating role of nutritional behaviors and nutritional status

**DOI:** 10.3389/fonc.2026.1862498

**Published:** 2026-09-15

**Authors:** Guiju Zhang, Cancan Du, Junwei Bai, Junmeng Li, Mengxiao Hou, Pengfei Yu, Yumei Tang

**Affiliations:** Department of Gastrointestinal Surgery, Henan Provincial People’s Hospital, Zhengzhou, China

**Keywords:** gastric cancer, health beliefs, nutritional behaviors, nutritional status, quality of life

## Abstract

**Background:**

Patients with gastric cancer often have reduced quality of life (QoL). This study aimed to examine whether nutritional behaviors and nutritional status serially mediate the association between health beliefs and QoL among patients with gastric cancer.

**Methods:**

This cross-sectional study included 274 patients with gastric cancer. Health beliefs, QoL, nutritional behaviors, and nutritional status were measured using validated instruments. Pearson correlation analysis was performed to examine bivariate relationships. Serial mediation analysis was conducted using structural equation modeling with bootstrap resampling (5,000 samples).

**Results:**

Health beliefs were positively correlated with QoL (*r* = 0.323, *P* < 0.01). The total effect of health beliefs on QoL was significant (effect = 0.595, *P* < 0.05). After including mediators, the direct effect remained significant (effect = 0.284, *P* < 0.05), accounting for 47.7% of the total effect. The indirect effect via nutritional behaviors alone was significant (effect = 0.150, *P* < 0.05), explaining 25.2% of the total effect. The indirect effect via nutritional status alone was also significant (effect = 0.103, *P* < 0.05), explaining 17.3% of the total effect. The serial indirect effect (health beliefs → nutritional behaviors → nutritional status → QoL) was significant (effect = 0.058, *P* < 0.05), accounting for 9.7% of the total effect. The model demonstrated excellent fit (*χ*²/*df* = 1.260, CFI = 0.992, RMSEA = 0.031, TLI = 0.989, GFI = 0.965, AGFI = 0.947).

**Conclusion:**

Health beliefs are positively associated with QoL in patients with gastric cancer, with evidence of independent and serial mediation by nutritional behaviors and nutritional status. These findings are compatible with the hypothesized theoretical model and support the development of theory-driven interventions for future testing in prospective studies.

## 1 Introduction

Gastric cancer remains a highly prevalent and lethal malignancy worldwide, imposing a substantial global health burden. According to GLOBOCAN 2022, it is the fifth most common cancer and the third leading cause of cancer-related death, with approximately 968,000 new cases and 660,000 deaths annually (1). Incidence varies geographically, peaking in Asia, South America, and Eastern Europe, and lowest in North America and Western Europe (2). Although incidence has declined in some regions due to improved dietary habits and H. pylori eradication, prognosis for advanced disease remains poor. Five-year survival ranges from roughly 13% (stage IV) to 85% (stage I) (3). The disease and its aggressive treatments, including gastrectomy, perioperative chemotherapy, and radiation, frequently cause debilitating symptoms such as early satiety, dysphagia, pain, fatigue, and weight loss, all of which profoundly impair patients’ physical, psychological, and social functioning (4–6). Consequently, health-related quality of life (QoL) has emerged as a critical patient-reported outcome in gastric cancer care, and identifying modifiable factors associated with QoL and elucidating their mechanisms is of paramount importance. Patients who have undergone gastrectomy and are receiving adjuvant or palliative chemotherapy constitute the most prevalent and clinically representative subgroup of gastric cancer patients actively managing the disease. Investigating the mechanistic pathways from cognitive factors to QoL in this population therefore carries strong clinical relevance and broad applicability.

Health beliefs, defined as individuals’ subjective perceptions of health threats and recommended behaviors as grounded in the Health Belief Model (HBM)-encompassing perceived susceptibility, severity, benefits, barriers, self-efficacy, and cues to action-are modifiable cognitive factors that shape health-related decision-making (7). In cancer care, these beliefs have been extensively linked to screening uptake, treatment adherence, and lifestyle modification (8, 9). Recent evidence indicates that positive health beliefs are associated with better QoL. For instance, higher cancer-coping self-efficacy has been linked to better multi-domain QoL in advanced gastric cancer patients, and psychosocial interventions modifying illness perceptions have been shown to reduce anxiety and improve global health status (10, 11). Based on this theoretical and empirical foundation, we propose that health beliefs among patients with gastric cancer are positively associated with QoL (H1).

Building on this foundation, the HBM provides a theoretical link from health beliefs to nutritional behaviors-eating practices such as meal frequency and dietary adherence. Patients who perceive high malnutrition susceptibility, acknowledge severe consequences, recognize benefits of dietary guidelines, and possess strong self-efficacy are more likely to adopt adaptive eating practices (12). In the context of gastric cancer, post-surgical anatomical alterations (e.g., reduced gastric capacity, dumping syndrome) impose strict dietary constraints, and while maladaptive habits can exacerbate weight loss and fatigue, adaptive nutritional behaviors can alleviate symptoms and improve functioning (13–16). Previous work has shown that gastric cancer survivors with stronger beliefs about nutrition’s role in recovery report more frequent protein-rich and small-meal intake, and that adherence to dietary recommendations is correlated with better QoL (6, 17–19). Therefore, we hypothesize that nutritional behaviors mediate the positive relationship between health beliefs and QoL (H2).

In addition to the behavioral pathway, nutritional status-reflecting the physiological balance between nutrient intake, absorption, and metabolic demands-represents a proximal physiological outcome closely linked to both health beliefs and QoL (20). Compromised nutritional status, manifesting as weight loss, low serum albumin, and sarcopenia, is highly prevalent in gastric cancer patients due to tumor-induced cachexia, post-surgical malabsorption, and chemotherapy side effects (21). This poor nutritional status is robustly associated with worse QoL across domains; a multicenter study of 5,310 older cancer patients found that malnourished individuals had significantly lower global health scores (22). Patients with adaptive health beliefs, especially high self-efficacy, are more likely to seek nutritional counseling and adhere to supplements, which has been associated with better physiological indicators such as higher serum albumin (19, 23). Given this, we propose that nutritional status independently mediates the positive relationship between health beliefs and QoL (H3).

Extending beyond these parallel pathways, a serial mediation pathway is theoretically plausible: health beliefs may first be associated with nutritional behaviors, which in turn relate to nutritional status, and ultimately to QoL. This sequential logic aligns with broader health behavior literature, where cognitive factors motivate behavioral change, behaviors influence physiological parameters, and improved physiology enhances subjective well-being (24). In gastric cancer, patients with adaptive beliefs adopt beneficial dietary practices that improve nutrient absorption and maintain muscle mass; better nutritional status then enables physical activity and reduces fatigue, thereby enhancing QoL (17, 25). Conversely, maladaptive beliefs may relate to poor behaviors, weight loss, and sarcopenia, impairing functioning and QoL. Emerging studies from upper gastrointestinal cancers provide preliminary support for these sequential links-for example, adherence to oral nutritional supplementation has been related to improved handgrip strength and QoL, and malnutrition has been independently associated with lower physical functioning scores (22, 23, 26, 27). However, no study has explicitly tested this serial mediating pathway in a single analytic model among gastric cancer patients. Addressing this gap would illuminate the mechanistic pathway from psychological cognition to tangible physiological and QoL outcomes, providing clear targets for sequential interventions. Therefore, we propose that nutritional behaviors and nutritional status serially mediate the positive relationship between health beliefs and QoL (H4).

In summary, gastric cancer patients experience significant QoL impairment from disease and treatment. Health beliefs are modifiable cognitive factors theoretically and empirically linked to QoL, and nutritional behaviors and status are two key explanatory variables. The HBM offers a coherent framework for understanding how health beliefs relate to dietary practices and physiological outcomes. This study proposes a serial mediation model in which health beliefs are associated with QoL first through nutritional behaviors and then through nutritional status. Testing this model may clarify the pathways from psychological cognition to clinical outcomes and inform the development of sequential, theory-driven interventions to improve QoL in gastric cancer patients. The hypothesized mediation model is shown in **Figure 1**.

**Figure 1:**
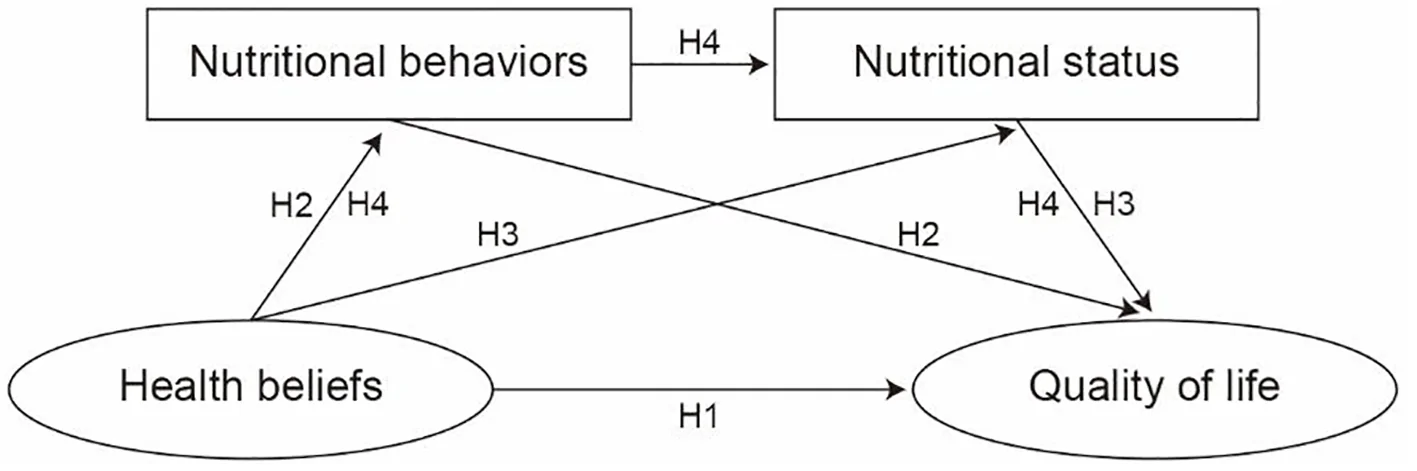
Hypothesized mediation model.

## 2 Methods

### 2.1 Study design

This cross-sectional observational study followed the Strengthening the Reporting of Observational Studies in Epidemiology (STROBE) guidelines. Data were collected from a convenience sample of gastric cancer patients at Henan Provincial People’s Hospital in China. All participants completed validated self-report questionnaires. The study protocol was approved by the Ethics Committee of Henan Provincial People’s Hospital (No. 2025160), and all procedures conformed to the ethical standards of the Declaration of Helsinki.

### 2.2 Participants

Eligible participants were patients with histologically confirmed gastric cancer who had undergone gastrectomy and were receiving adjuvant or palliative chemotherapy. Inclusion criteria: (a) age ≥ 18 years, (b) ability to read and understand the questionnaires independently or with assistance, and (c) provision of written informed consent. Exclusion criteria: (a) other concurrent malignancies, (b) severe cognitive impairment or psychiatric disorders precluding reliable self-report, (c) active critical complications (e.g., gastrointestinal bleeding, perforation, or obstruction requiring emergency intervention), and (d) exclusive enteral or parenteral nutrition without oral intake.

A convenience sampling strategy was adopted. Based on guidelines for testing indirect effects in a serial mediation model (80% power, α = 0.05, small-to-medium effect size), a minimum of 200–250 participants were required (28). To account for incomplete data, we aimed to recruit 300 eligible patients. Recruitment was conducted from April 1, 2024, to December 31, 2025, at the oncology and gastrointestinal surgery outpatient clinics and inpatient wards of the participating hospitals. Trained research assistants screened consecutive patients, explained the study, and obtained written informed consent. For inpatients, questionnaires were completed in a quiet room on the ward during a stable period of hospitalization, defined as at least 48 hours after any acute event. For outpatients, questionnaires were completed in a designated quiet area of the outpatient clinic prior to scheduled chemotherapy sessions or follow-up visits. Clinical and demographic data were extracted from electronic medical records. For quality control, all questionnaires were checked for completeness on-site; incomplete forms were returned to participants for immediate supplementation. After this procedure, the total missing data rate was below 5%. Any items that still remained missing were addressed via multiple imputation. Data were double-entered and verified by two independent researchers to minimize entry errors. A total of 300 questionnaires were distributed, 287 were returned, and 274 were valid, yielding a valid response rate of 91.3%.

### 2.3 Demographic and clinical characteristics

Demographic and clinical data were collected using a standardized form and verified through medical record review. Variables included age, gender, education level, marital status, BMI, cancer stage, number of chemotherapy cycles, chemotherapy intent (adjuvant/palliative), and time from surgery to questionnaire completion. Surgical procedure was categorized as total gastrectomy or subtotal gastrectomy based on operative records. Smoking status was classified as “ever smoker” (including current and former smokers) or “never smoker” according to patient self-report and medical history. Cancer stage was classified according to the eighth edition of the AJCC staging system (29). The number of chemotherapy cycles was recorded as a continuous variable and further categorized for descriptive analysis (≤3, 4-6, and ≥7 cycles) (30). All data were extracted from electronic medical records by trained research assistants and checked for completeness.

### 2.4 Health beliefs

Health beliefs were measured using an 18-item scale adapted from Champion’s Health Belief Model Scale (31). The adaptation process involved a review of the original items by an expert panel of two oncologists, three oncology nurses, and two nutritionists, who revised the wording to align with nutrition-related beliefs in gastric cancer care, and a subsequent pilot test of the adapted Chinese version on 30 gastric cancer patients to assess face validity and comprehension. The adapted scale was contextualized to the Chinese healthcare setting and has shown satisfactory reliability and validity in previous research (32). The instrument comprises five dimensions: perceived susceptibility, perceived severity, perceived benefits, perceived barriers, and self-efficacy. Items are rated on a 5-point Likert scale ranging from 1 (completely disagree) to 5 (completely agree). Higher dimension scores indicate stronger health beliefs. In the present study, the Cronbach’s α for the total scale was 0.802.

### 2.5 QoL

QoL was assessed with the Functional Assessment of Cancer Therapy-General (FACT-G) (33). This 27-item instrument encompasses four domains: physical, functional, emotional, and social/family well-being. Responses are scored on a 5-point Likert scale from 0 (not at all) to 4 (very much), with five items requiring reverse scoring. The total score ranges from 0 to 108, where higher values denote superior QoL. The validated Chinese version of the FACT-G was employed (34). In this study, the Cronbach’s α for the total scale was 0.834.

### 2.6 Nutritional behaviors

Nutritional behaviors were evaluated using the Nutritional Behavior Subscale of the Digestive Cancer Patients Nutrition Knowledge, Attitude, and Practice Questionnaire (35). This subscale has been validated among Chinese gastrointestinal cancer patients (35). It comprises eight items addressing dietary patterns and nutrition-related self-management behaviors relevant to gastric cancer. Responses are recorded on a 5-point Likert scale, with elevated scores reflecting more favorable nutritional behaviors. The Cronbach’s α for this subscale in the current sample was 0.821.

### 2.7 Nutritional status

Nutritional status was determined using the Scored Patient-Generated Subjective Global Assessment (PG-SGA) (36), a well-established and validated instrument for nutritional evaluation in oncology populations. Total PG-SGA scores range from 0 to 35, with higher scores signifying poorer nutritional status and an increased need for nutritional intervention. The PG-SGA has exhibited robust validity and reliability in the assessment of nutritional status among Chinese patients with gastric cancer (37). In the present investigation, the Cronbach’s α for the PG-SGA was 0.872.

### 2.8 Statistical analysis

Analyses were performed using SPSS 27.0 and Amos 26.0. Categorical variables were summarized as frequencies and percentages, whereas continuous variables were expressed as means ± standard deviations (SD) following assessment of distribution normality. Normality was assessed using the Shapiro-Wilk test and by examining skewness and kurtosis values. Group comparisons of QoL scores across demographic and clinical characteristics were conducted using independent-samples t-tests and one-way analysis of variance (ANOVA). Bivariate relationships among health beliefs, nutritional behaviors, nutritional status, and QoL were examined via Pearson correlation coefficients. To assess the potential influence of common method bias, we conducted Harman’s single-factor test using exploratory factor analysis. A single factor accounting for more than 50% of the total variance would indicate the presence of substantial common method bias (38).

The hypothesized serial mediation model was evaluated within a structural equation modeling framework employing maximum likelihood estimation. In the SEM model, health beliefs and QoL were specified as latent variables, with their respective subdomain scores serving as indicators. Nutritional behaviors and nutritional status were specified as observed variables. Additionally, all covariates were included as observed variables and adjusted for in the model. For latent variables, factor loadings, standard errors, and composite reliability (CR) and average variance extracted (AVE) values were calculated to assess construct validity. Indirect effects were quantified through bias-corrected bootstrapping with 5,000 resamples, yielding 95% confidence intervals (CIs). Model adequacy was assessed using the following fit indices and associated cut-off criteria: *χ*²/*df* < 3, comparative fit index (CFI) > 0.90, Tucker-Lewis index (TLI) > 0.90, root mean square error of approximation (RMSEA) < 0.08, goodness-of-fit index (GFI) > 0.90, and adjusted goodness-of-fit index (AGFI) > 0.90 (39). All hypothesis tests were two-tailed, and statistical significance was defined as *P* < 0.05.

## 3 Results

### 3.1 Sample characteristics

A total of 274 patients with gastric cancer were included in the final analysis. The median time from surgery to questionnaire completion was 8.0 (1.5-18.0) months. Demographic and clinical characteristics of the sample, along with comparisons of QoL scores across subgroups, are presented in **Table 1**. The mean age was 57.7 ± 12.6 years, and the majority were male (70.1%), married (83.6%), and had completed less than high school education (56.9%). Regarding clinical characteristics, 35.8% were ever smokers, 61.7% of participants had a BMI within the normal range (18.5-23.9 kg/m²), 42.3% were diagnosed with stage III disease, 45.6% had received 4–6 cycles of chemotherapy, and 37.6% underwent total gastrectomy. In addition, QoL scores did not differ significantly across any demographic or clinical subgroup (all *P* > 0.05).

**Table 1:** Comparison of participants’ general demographic characteristics and quality of life score in different subgroups (n=274).

Variable	n (%)	Mean ± SD	*t/F*	*P*
Age (years)			0.105 ^B^	0.900
18-44	40 (14.6)	63.0 ± 18.2		
45-59	118 (43.1)	62.4 ± 18.2		
≥60	116 (42.3)	61.6 ± 18.7		
Gender			1.289 ^A^	0.198
Female	82 (29.9)	64.4 ± 17.2		
Male	192 (70.1)	61.2 ± 18.8		
Education level			-0.154 ^A^	0.878
<High School	156 (56.9)	62.0 ± 17.4		
≥High School	118 (43.1)	62.4 ± 19.6		
Marital status			1.647 ^B^	0.194
Single	32 (11.7)	67.5 ± 15.1		
Married	229 (83.6)	61.3 ± 18.8		
Divorce/Widowhood	13 (4.7)	63.6 ± 17.3		
Smoking			1.024 ^A^	0.307
Ever smoker	98 (35.8)	61.0 ± 18.5		
Never smoker	176 (64.2)	63.2 ± 18.3		
BMI			0.089 ^B^	0.915
<18.5	29 (10.6)	62.9 ± 20.0		
18.5-23.9	169 (61.7)	61.8 ± 18.1		
≥24.0	76 (27.7)	62.7 ± 18.6		
Cancer stage			0.236 ^B^	0.871
I	41 (15.0)	62.9 ± 17.4		
II	56 (20.4)	63.8 ± 18.0		
III	116 (42.3)	61.5 ± 18.9		
IV	61 (22.3)	61.5 ± 18.6		
Chemotherapy cycles			0.044 ^B^	0.957
≤3	105 (38.3)	61.8 ± 18.4		
4-6	125 (45.6)	62.4 ± 18.5		
≥7	44 (16.1)	62.6 ± 18.2		
Chemotherapy intent			0.251 ^A^	0.802
Adjuvant	196 (71.5)	62.4 ± 18.2		
Palliative	78 (28.5)	61.8 ± 19.0		
Surgical procedure			1.887 ^A^	0.060
Total gastrectomy	103 (37.6)	59.5 ± 18.9		
Subtotal gastrectomy	171 (62.4)	63.7 ± 17.9		

SD, Standard deviation; BMI, Body mass index. A, independent-samples t-tests; B, one-way ANOVA.

### 3.2 Descriptive statistics and correlations

Normality tests indicated that QoL scores, health beliefs total scores, nutritional behaviors scores, and PG-SGA scores were approximately normally distributed (all *P* > 0.05). Means, standard deviations, and bivariate correlations among the study variables are displayed in **Table 2**. The mean total QoL score was 62.2 ± 18.4. Health beliefs total score was positively correlated with QoL (*r* = 0.323, *P* < 0.01), as were all health belief dimensions including perceived susceptibility (*r* = 0.279), perceived severity (*r* = 0.286), perceived benefits (*r* = 0.334), and self-efficacy (*r* = 0.305, all *P* < 0.01), whereas perceived barriers was negatively correlated with QoL (*r* = -0.304, *P* < 0.01). Nutritional behaviors were positively associated with QoL (*r* = 0.420, *P* < 0.01) and with health beliefs total score (*r* = 0.390, *P* < 0.01). Nutritional status, as measured by the Scored PG-SGA where higher scores indicate poorer nutritional status, was negatively correlated with QoL (*r* = -0.467, *P* < 0.01), health beliefs total score (*r* = -0.296, *P* < 0.01), and nutritional behaviors (*r* = -0.356, *P* < 0.01).

**Table 2:** Correlation analysis of study variables.

Variable	Mean ± SD	Health beliefs	Perceived susceptibility	Perceived severity	Perceived benefits	Perceived barriers	Self-efficacy	Nutritional behaviors	Nutritional status	Quality of life
Health beliefs	53.6 ± 11.3	1.000	–	–	–	–	–	–	–	–
Perceived susceptibility	12.0 ± 4.0	0.835^**^	1.000	–	–	–	–	–	–	–
Perceived severity	13.0 ± 4.2	0.838^**^	0.577^**^	1.000	–	–	–	–	–	–
Perceived benefits	8.8 ± 3.0	0.813^**^	0.644^**^	0.613^**^	1.000	–	–	–	–	–
Perceived barriers	8.0 ± 2.8	-0.446^**^	-0.481^**^	-0.538^**^	-0.474^**^	1.000	–	–	–	–
Self-efficacy	11.9 ± 3.9	0.840^**^	0.627^**^	0.677^**^	0.613^**^	-0.569^**^	1.000	–	–	–
Nutritional behaviors	28.0 ± 6.7	0.390^**^	0.349^**^	0.352^**^	0.368^**^	-0.320^**^	0.341^**^	1.000	–	–
Nutritional status	10.6 ± 6.5	-0.296^**^	-0.279^**^	-0.237^**^	-0.218^**^	0.205^**^	-0.296^**^	-0.356^**^	1.000	–
Quality of life	62.2 ± 18.4	0.323^**^	0.279^**^	0.286^**^	0.334^**^	-0.304^**^	0.305^**^	0.420^**^	-0.467^**^	1.000

^*^ *P* < 0.05, ^**^P < 0.01. A higher PG-SGA score indicates worse nutritional status.

### 3.3 Mediation analysis

The Harman’s single-factor test indicated that the first unrotated factor accounted for 28.6% of the total variance. The hypothesized serial mediation model was tested using structural equation modeling. The model demonstrated excellent fit to the data: *χ*²/*df* = 1.260, CFI = 0.992, TLI = 0.989, RMSEA = 0.031, GFI = 0.965, AGFI = 0.947. The latent variable measurement models demonstrated adequate construct validity. For health beliefs, factor loadings ranged from 0.71 to 0.89 (all *P* < 0.001), with CR = 0.91 and AVE = 0.68. For QoL, factor loadings ranged from 0.74 to 0.86 (all *P* < 0.001), with CR = 0.88 and AVE = 0.65. The standardized path coefficients for the final model are illustrated in **Figure 2**.

**Figure 2:**
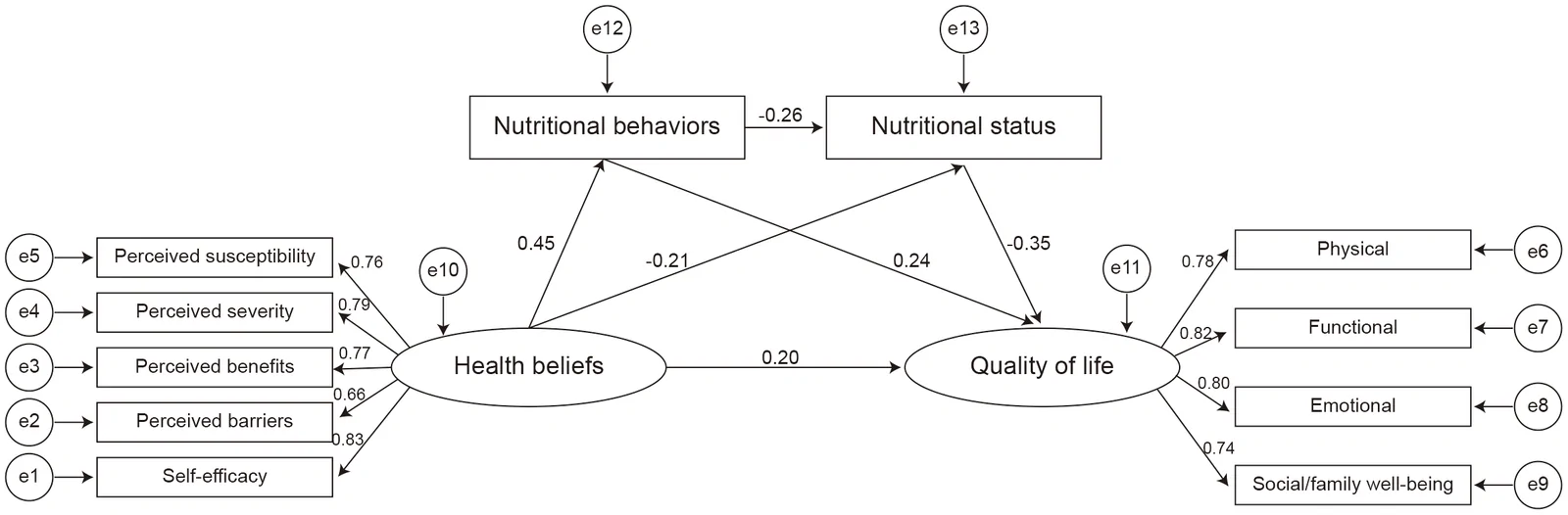
Chain mediation structural equation model. Standardized path coefficients are displayed. A higher PG-SGA score indicates worse nutritional status. Adjusted age, gender, education level, marital status, smoking, BMI, cancer stage, chemotherapy cycles, and surgical procedure.

As presented in **Table 3**, the total effect of health beliefs on QoL was significant (effect = 0.595, *P* < 0.05). After introducing nutritional behaviors and nutritional status as mediators, the direct effect remained significant (effect = 0.284, *P* < 0.05), accounting for 47.7% of the total effect. Regarding the specific indirect effects, all three hypothesized mediation pathways were statistically significant. The indirect effect via nutritional behaviors alone (Ind1: health beliefs → nutritional behaviors → QoL) was 0.150 (*P* < 0.05), accounting for 25.2% of the total effect. The indirect effect via nutritional status alone (Ind2: health beliefs → nutritional status → QoL) was 0.103 (*P* < 0.05), explaining 17.3% of the total effect. Furthermore, the serial indirect effect (Ind3: health beliefs → nutritional behaviors → nutritional status → QoL) was 0.058 (*P* < 0.05), contributing 9.7% to the total effect.

**Table 3:** Analysis of the mediating effect of nutritional behaviors and nutritional status.

Mediating path	Effect size	BootSE	Boot (95% CI)	*P*	Proportion mediated (%)
Total effect	0.595	0.103	0.404-0.807	<0.001	100.0
Direct effect	0.284	0.097	0.102-0.485	0.002	47.7
Mediating effect					
Ind1	0.150	0.044	0.073-0.241	<0.001	25.2
Ind2	0.103	0.040	0.028-0.182	0.007	17.3
Ind3	0.058	0.022	0.022-0.109	0.001	9.7

A higher PG-SGA score indicates worse nutritional status.

Direct effect: Health beliefs→Quality of life (i.e., the remaining direct association not mediated by the two mediators);

Ind1: Health beliefs→Nutritional behaviors→Quality of life;

Ind2: Health beliefs→Nutritional status→Quality of life;

Ind3: Health beliefs→Nutritional behaviors→Nutritional status→Quality of life.

## 4 Discussion

This study investigated the relationship between health beliefs and QoL in patients with gastric cancer, and examined the serial mediating roles of nutritional behaviors and nutritional status within the HBM. Results showed that health beliefs were positively associated with QoL, supporting Hypothesis 1. Additionally, nutritional behaviors and nutritional status each independently showed mediating effects in this relationship, confirming Hypotheses 2 and 3. Importantly, a serial mediation pathway was identified: health beliefs were associated with QoL first through nutritional behaviors, then through nutritional status, thereby supporting Hypothesis 4. These findings offer preliminary strategies for improving QoL in this population.

Our study demonstrated a positive association between health beliefs and QoL. Under the HBM, patients who reported higher susceptibility to and severity of malnutrition-related complications, recognized benefits of dietary self-management, reported fewer barriers to adherence, and had greater self-efficacy also reported better QoL (40). In the present sample, perceived benefits and self-efficacy showed the strongest correlations with QoL among the five HBM dimensions. Notably, QoL scores did not differ significantly across demographic or clinical subgroups (age, gender, cancer stage, chemotherapy cycles), suggesting that health beliefs represent a consistently relevant factor associated with QoL independent of disease severity or treatment burden. This finding suggests the clinical importance of considering patients’ cognitive representations of health threats, even among those with advanced disease.

Nutritional behaviors showed a mediating effect in the health beliefs-QoL association, with an indirect effect accounting for 25.2% of the total effect. This finding aligns with the HBM premise that cognitive antecedents motivate behavioral enactment (12). Gastric cancer patients with adaptive health beliefs were more likely to adopt beneficial dietary practices, such as small frequent meals, protein-rich food choices, thorough chewing, and avoidance of concentrated sweets. These behaviors were associated with reduced post-gastrectomy symptoms (early satiety, dumping syndrome, reflux) and were thereby associated with better physical and functional well-being (13). This pathway suggests nutritional behaviors as a critical mechanism translating psychological resources into improved daily functioning and subjective QoL, consistent with broader health behavior literature on dietary adherence in chronic conditions (12, 41). Clinically, interventions that enhance self-efficacy for dietary self-management and reduce perceived barriers to adherence might promote adaptive eating behaviors and, subsequently, better QoL.

Nutritional status independently showed a mediating effect in the relationship, accounting for 17.3% of the total effect. Patients with stronger health beliefs also had better nutritional status (lower PG-SGA scores), which in turn was associated with higher QoL. This aligns with prior research demonstrating strong associations between malnutrition and impaired QoL in older cancer patients (42). Health beliefs may relate to nutritional status through multiple potential mechanisms: patients with high self-efficacy and perceived benefits of nutrition-focused care may be more likely to seek dietary counseling, adhere to oral nutritional supplements, and monitor weight regularly, thereby preventing or mitigating malnutrition (23).

The serial mediation pathway (health beliefs → nutritional behaviors → nutritional status → QoL) was also significant, accounting for 9.7% of the total effect. This sequential mechanism, although theoretically coherent, has not been previously tested in gastric cancer. Patients with adaptive beliefs were more likely to adopt specific dietary practices that were associated with improved nutrient absorption and energy balance, thereby stabilizing nutritional status. Improved nutritional status was in turn associated with better physical functioning, reduced fatigue, and enhanced overall QoL (43). The significant serial indirect effect is consistent with a conceptually ordered sequence in which nutritional behaviors and nutritional status operate sequentially rather than as parallel mediators, conceptualizing the full pathway from cognition to behavior to physiology to subjective outcome within a single analytic framework (41). The excellent model fit indices (*χ*²/*df* = 1.260, CFI = 0.992, TLI = 0.989, RMSEA = 0.031, GFI = 0.965, AGFI = 0.947) support the robustness of this theoretical model.

This study offers several contributions. It is among the first to empirically test the serial mediation pathway from health beliefs to QoL via nutritional behaviors and nutritional status in gastric cancer patients. Although the hypothesized sequence has theoretical grounding in health behavior literature, prior studies in this population have mainly examined direct or single-mediator effects. Our study integrates these pathways into one framework, offering a more comprehensive mechanistic account of how cognitive factors relate to patient-reported outcomes through behavioral and physiological intermediates. Quantifying each pathway’s proportion of the total effect (behavioral 25.2%, physiological 17.3%, serial 9.7%) provides preliminary evidence to inform intervention prioritization. Additionally, applying the HBM, traditionally used for preventive behaviors, to post-treatment QoL illustrates its broader utility. Nevertheless, these results provide an empirical basis for theory-driven hypothesis generation and underscore the need to address behavioral and physiological mechanisms in cancer care. Given the cross-sectional design, findings are hypothesis-generating, not confirmatory. Prospective studies are required to establish temporal precedence and test the proposed causal sequence.

Several limitations warrant consideration. The cross-sectional design precludes causal inference and cannot rule out reverse causality; Convenience sampling from a single geographic region in China may constrain generalizability, given that health beliefs and dietary practices are shaped by cultural norms, healthcare system attributes, and socioeconomic factors; The combined inclusion of adjuvant and palliative chemotherapy patients represents clinical heterogeneity; Except for nutritional status, measures relied on self-report instruments susceptible to recall bias and social desirability effects. Additionally, the adapted scale used to measure health beliefs has not been fully psychometrically tested in this specific gastric cancer population, and its further validation in larger and more diverse samples is warranted; Unmeasured confounders such as depression, anxiety, social support, and socioeconomic status may independently influence health beliefs, nutritional behaviors, and QoL and should be accounted for in subsequent research; Critically, unmeasured post-gastrectomy sequelae (e.g., early satiety, dumping syndrome, reflux, cancer-related fatigue) may constrain dietary intake and QoL, potentially acting as confounders or competing mediators that could inflate the observed associations between health beliefs and nutritional outcomes; The timing since surgery varied across participants, and nutritional challenges may differ between the early postoperative period and long-term survivorship. Future investigations should address these limitations through multicenter longitudinal designs with comprehensive assessment of psychological, social, and clinical confounders.

## 5 Conclusion

In conclusion, this study provides evidence that health beliefs are positively associated with QoL in patients with gastric cancer, and that this relationship is statistically consistent with independent and serial mediation by nutritional behaviors and nutritional status. These findings highlight the importance of assessing and addressing patients’ health beliefs as part of comprehensive cancer care. They also suggest that theory-driven interventions targeting cognitive determinants of dietary behavior may represent a promising strategy for improving nutritional outcomes and QoL, although prospective and randomized studies are needed to test these hypotheses.

## Data Availability

The raw data supporting the conclusions of this article will be made available by the authors, without undue reservation.
